# CRISPR/Cas9-Mediated Multi-Allelic Gene Targeting in Sugarcane Confers Herbicide Tolerance

**DOI:** 10.3389/fgeed.2021.673566

**Published:** 2021-07-08

**Authors:** Mehmet Tufan Oz, Angelika Altpeter, Ratna Karan, Aldo Merotto, Fredy Altpeter

**Affiliations:** ^1^Agronomy Department, Plant Molecular and Cellular Biology Program, Genetics Institute, University of Florida, IFAS, Gainesville, FL, United States; ^2^DOE Center for Advanced Bioenergy and Bioproducts Innovation, Gainesville, FL, United States

**Keywords:** homologous recombination, CRISPR/Cas9, sugarcane (*Saccharum* hybrid complex), genome editing, gene targeting, homology directed repair

## Abstract

Sugarcane is the source of 80% of the sugar and 26% of the bioethanol produced globally. However, its complex, highly polyploid genome (2*n* = 100 – 120) impedes crop improvement. Here, we report efficient and reproducible gene targeting (GT) in sugarcane, enabling precise co-editing of multiple alleles *via* template-mediated and homology-directed repair (HDR) of DNA double strand breaks induced by the programmable nuclease CRISPR/Cas9. The evaluation of 146 independently transformed plants from five independent experiments revealed a targeted nucleotide replacement that resulted in both targeted amino acid substitutions W574L and S653I in the acetolactate synthase (ALS) in 11 lines in addition to single, targeted amino acid substitutions W574L or S653I in 25 or 18 lines, respectively. Co-editing of up to three ALS copies/alleles that confer herbicide tolerance was confirmed by Sanger sequencing of cloned long polymerase chain reaction (PCR) amplicons. This work will enable crop improvement by conversion of inferior alleles to superior alleles through targeted nucleotide substitutions.

## Introduction

Sugarcane (*Saccharum* spp. hybrid) is the source of 80% of the world's sugar and 26% of its bioethanol, and it is an exceptionally productive crop due to its superior light conversion and water- and nitrogen-use efficiencies (Byrt et al., 2011). The genome of sugarcane is the most complex of any domesticated agricultural species (2*n* = 100–120) (Piperidis and D'Hont, 2020). Modern sugarcane cultivars are derived from hybridization between *Saccharum officinarum* (2*n* = 80, *x* = 10) and *Saccharum spontaneum* (2*n* = 40–128, *x* = 8); these are responsible for high sugar content and stress tolerance or vigor, respectively (Piperidis and D'Hont, 2020). Elevated sugar production was achieved by backcrossing the hybrid to *S*. *officinarum*. The resulting cultivars are aneuploid, highly heterozygous, and highly polyploid, with 100–120 chromosomes. Most chromosomes are derived from *S*. *officinarum*, depending on the cultivar, with 10–20% originating from *S*. *spontaneum* and ~10% from interspecific recombinants (D'Hont et al., 1996). Modern cultivars typically have 12 copies of each of the first four basic chromosomes, while parent species tend to differ in those basic chromosomes. One to four of these copies correspond to entire *S*. *spontaneum* chromosomes or interspecific recombinant chromosomes. In addition, inter-chromosomal translocations are also present (Garsmeur et al., 2018; Piperidis and D'Hont, 2020). Unsurprisingly, elite cultivars require vegetative propagation to maintain their quality and agronomic performance. Cultivar development by conventional breeding must overcome the challenges of photoperiod sensitivity in floral induction, lack of pollen fertility, and synchrony of flowering in most parental sugarcane clones (Horsley and Zhou, 2013).

Genome editing with sequence-specific nucleases is revolutionizing crop breeding (Voytas and Gao, 2014; Zhang et al., 2018) and has promising applications for sugarcane and other vegetatively propagated polyploid crops with complex genomes (Jung and Altpeter, 2016; Weeks, 2017). DNA cleavage through sequence-specific nucleases, including CRISPR/Cas9, is followed by the use of cellular repair mechanisms, including non-homologous end joining (NHEJ) or homology-directed repair (HDR), to rectify double-strand breaks (DSBs). Non-homologous end joining enables the construction of knockout alleles through frameshift mutations. By contrast, HDR-mediated gene targeting (GT) allows the introduction of precise genetic modifications, including single-nucleotide substitutions, gene replacements, and large insertions (Huang and Puchta, 2019). Gene targeting requires recombination with a repair template with homology to the break site. The repair template may be the sister chromatid or a co-introduced targeting vector, containing a desired sequence modification for incorporation into the break site (Puchta, 1998; Bortesi and Fischer, 2015; Que et al., 2019).

Homology-directed repair-mediated GT in plant genomes remains a challenge, resulting in a small number of successful studies (Chen et al., 2019; Sedeek et al., 2019). In contrast to knock-outs with up to a 100% mutation frequency (Brooks et al., 2014), precise gene replacement frequencies are typically in the range of 0.1 to a few percent, with large, targeted insertions being the most challenging (Huang and Puchta, 2019; Mao et al., 2019). Because GT is typically an inefficient process, mutations that confer a selectable phenotype, such as herbicide resistance, have been favored as initial targets for recovering the events that caused them (Shukla et al., 2009; Svitashev et al., 2015, 2016; Butler et al., 2016; Sun et al., 2016). The acetolactate synthase (ALS) enzyme catalyzes the biosynthesis of essential branched-chain amino acids (Smith et al., 1989) and is strongly inhibited by several herbicides, such as sulfonylureas, imidazolinones, triazolopyrimidines, pyrimidinyloxybenzoates, and sulfanilamide-carbonyl-thiazolidinones (Smith et al., 1989; Shaner and O'Connor, 1991; Devine et al., 1992; Duggleby et al., 2008; Powles and Yu, 2010). Resistance to ALS-inhibiting herbicides is controlled by specific mutations of the *ALS* gene at amino acid positions Ala122, Pro197, Ala205, Asp376, Arg377, Trp574, Ser653, and Ser654 (Tan et al., 2006; Li et al., 2008; Merotto et al., 2009; Rodríguez-Suárez et al., 2009). One of these mutations, Trp574, confers high-level resistance and cross-resistance to all ALS-inhibiting herbicides (Tan et al., 2006). The Pro197 mutation results in the greatest tolerance to sulfonylureas. Mutations at Ala122, Ala205, and Ser653 provide resistance to imidazolinones (Tan et al., 2006). Mutated herbicide-resistant *ALS* alleles are semi-dominant, and increased resistance has been ascribed to two or more resistance-conferring codons and/or resistance genes or alleles in a given genotype (Pozniak and Hucl, 2004; Shimizu et al., 2005).

Here, we report efficient and reproducible multi-allelic GT in sugarcane. We also provide an evaluation of two alternative sgRNAs used alone or in combination for altering one or two codons (W574L and/or S653I) that are known to confer herbicide tolerance. We also compared HDR frequency following the biolistic delivery of different quantities of the repair template.

## Materials and Methods

### Construction of Plasmid Vectors Carrying DNA-Editing Tools

Five plasmids carrying various DNA elements were constructed (Supplementary Figure 1, Supplementary Table 1). *Cas9* from *Streptococcus pyogenes* was codon-optimized for sugarcane and custom-synthesized, including nuclear localization signals from SV40 and nucleoplasmin, by GENEWIZ, Inc. (South Plainfield, NJ, USA). The selectable marker *neomycin phosphotransferase* II (*npt*II) and codon-optimized *Cas9* (*coCas9*) were under the transcriptional control of cauliflower mosaic virus 35S RNA (CaMV35S) promoter with a 70 kDa heat-shock protein (HSP70) intron and CaMV35S terminator or *Arabidopsis thaliana* heat-shock protein (AtHSP) terminator, respectively. Benchling (https://www.benchling.com) was used for the gRNA design process. The sgRNAs that direct cleavage near amino acid residue 574 (sgRNA1) and 653 (sgRNA2) were placed under the transcriptional control of U6 promoter from *Oryza sativa*. The 20 bp target sequences of sgRNA1 and sgRNA2 are 5′tcactgggaggttctcaatt and 5′gtcaaagaaaggcagggagg, respectively. The sgRNA constructs with U6 promoters were custom-synthesized by GENEWIZ. A repair template (Supplementary Table 2) was designed based on the sugarcane *ALS* sequence, with nucleotide modifications to introduce W574L and S653I, along with two modified PAM sites (PAM1 and PAM2) to prevent cleavage of the template by sgRNA1 or sgRNA2. Homology arms of 1,007 bp at the 5′-end and 447 bp at the 3′-end relative to the targeted DSBs were included in the design of the 1,833 bp double-stranded DNA template custom-synthesized by GENEWIZ.

### Cas9 *in vitro* Cleavage Assay

The targeting efficiency of the sgRNAs was investigated *in vitro* using a commercial Cas9 protein (PNA Bio, CA). A 1,174 bp target region of the *ALS* gene was amplified using Q5 high-fidelity DNA polymerase (NEB, MA) with the primer pair UP5 and DO1 (Supplementary Table 3) in a conventional polymerase chain reaction (PCR). The amplicon was isolated using gel electrophoresis and purified using the Monarch® DNA gel extraction kit (NEB). Two sgRNAs were transcribed *in vitro* using the HiScribe™ T7 quick high-yield RNA synthesis kit (NEB) from a DNA template generated with PCR. For this approach, a T7 promoter was fused upstream of the sgRNAs, using Q5 DNA polymerase with primer pair C1F and CR for sgRNA1 and primer pair C2F and CR for sgRNA2. After DNase I (RNase-free) treatment, synthesized RNA was purified through phenol:chloroform extraction followed by ethanol precipitation. An *in vitro* cleavage assay was performed at 37°C overnight in the presence of 450 ng target *ALS* fragment, 150 ng Cas9 protein, 300 ng sgRNA, and 100 μg/mL BSA buffered with Buffer 3 (NEB). Cleavage products were isolated using agarose gel electrophoresis to determine the sgRNA targeting efficiency.

### Generation of Genome-Edited Sugarcane

Plasmids carrying expression cassettes and donor template were introduced into sugarcane callus through biolistic gene transfer for indirect embryogenesis, as described earlier (Taparia et al., 2012). The amounts of DNA used per shot and the molar ratios of editing components are shown in Table 1. For treatments 1, 2, 3, and 5, 1.5 ng per kilobase DNA per shot (ng/kb and shot) was used (Wu et al., 2015). For treatment 4, 0.5 ng was used. The editing components were precipitated onto 1.8 mg gold particles using a protocol described previously (Sandhu and Altpeter, 2008). Approximately 35 calli per shot were placed without gaps to cover a circular target area 30 mm in diameter (Supplementary Figure 2B). Following biolistic gene transfer and callus selection with 20 mg/L Geneticin, calli were transferred to regeneration media containing 90.5 μg/L bispyribac sodium (BS) for shoot elongation for 60 days, followed by rooting on media without BS and growth regulators (Supplementary Figure 2). The targeted mutations W574L and S653I in the ALS gene confer resistance to several herbicides, including BS, as previously demonstrated in sugarcane with a transgenic approach (Dermawan et al., 2016).

**Table 1:** Frequency of allele replacement with homology-directed repair (HDR) following five different treatments for biolistic delivery of genome editing components.

**Treatment**	**Plasmid DNA amount (ng/kb and shot)**					**Shots**	**Lines PCR + for *npt*II**	**Lines after BS herbicide selection**	**Number of lines with intended mutations**			
	***Cas9***	***npt*II**	**gRNA1**	**gRNA2**	**Repair template**				**W574L only (%)^†^**	**S653I only (%)^†^**	**W574L and S653I (%)^†^**	**W574L and/or S653I (%)^†^**
1	1.5	1.5	1.5	0.0	6.0	10	84	28	8 (9.5%)	4 (4.8%)	1 (1.2%)	13 (15.5%)
2	1.5	1.5	0.0	1.5	6.0	10	91	27	7 (7.7%)	3 (3.3%)	6 (6.6%)	16 (17.6%)
3	1.5	1.5	1.5	1.5	6.0	10	97	25	5 (5.2%)	3 (3.1%)	1 (1.0%)	9 (9.3%)
4	0.5	0.5	0.5	0.5	2.0	10	102	32	4 (3.9%)	6 (5.9%)	3 (2.9%)	13 (12.7%)
5	1.5	1.5	1.5	1.5	12.0	10	93	34	1 (1.1%)	2 (2.2%)	0 (0%)	3 (3.2%)
**Total**							**467**	**146**	**25 (5.4%)**	**18 (3.9%)**	**11 (2.4%)**	**54 (11.6%)**

*Number of lines with intended mutations W574L and/or S653I at target locations in sugarcane ALS are shown. Introduction of mutations with HDR were determined using a TaqMan® probe-based genotyping assay. Cas9, codon optimized CRISPR-associated gene 9; nptII, neomycin phosphotransferase II; gRNA, single guide RNA; ALS, acetolactate synthase; BS, bysparibac sodium*.

† *Frequencies are calculated as percentage of lines with intended mutations W574L and/or S653I in lines positive for nptII and prior selection with the herbicide bysparibac sodium (BS). All lines that regenerated from media containing BS were analyzed*.

### Nucleic Acid Isolation and PCR

Following Geneticin selection and again following regeneration on culture media containing BS, approximately 0.1–0.2 g tissue was sampled from each line and frozen in liquid nitrogen. The frozen tissue was ground using TissueLyser II (Qiagen, Germany). Genomic DNA was extracted using cetyltrimethylammonium bromide as described earlier (Murray and Thompson, 1980). DNA was dissolved in 50–100 μl nuclease-free water and quantified using NanoDrop™ One (Thermo Fisher Scientific, MA, USA). The presence of transgenes in genomic DNA extracts was investigated using PCR with gene-specific primers (Supplementary Table 3).

### Verification of Target Mutations

**Figure 1:**
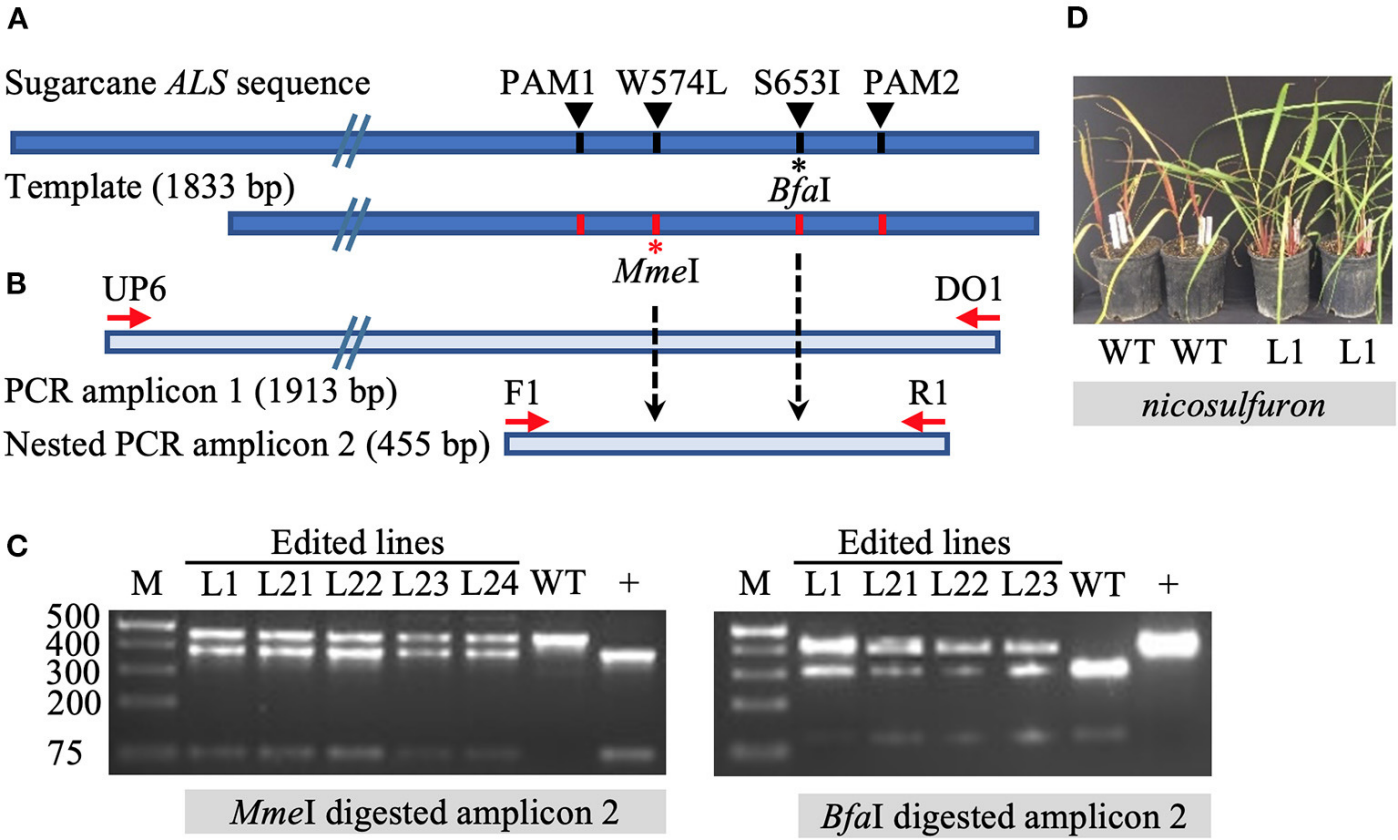
Identification of gene targeting in sugarcane by restriction endonuclease assays and herbicide application. **(A)** Repair template compared to sugarcane acetolactate synthase (*ALS*) gene. The 1,833 bp template carries four nucleotide substitutions, one at the codon positions W574L (introducing the *Mme*I recognition site) and S653I (eliminating *Bfa*I recognition site), as well as two modified PAM sites (PAM1 and PAM2) (preventing template cleavage by Cas9 complexed with sgRNA1 or sgRNA2). Amino acid residue numbering in the sugarcane *ALS* gene follows the *Arabidopsis* nomenclature. **(B)** Schematic representation of primer annealing positions. Primers DO1 and UP6 were used to amplify a 1,913 bp fragment. Using primer UP6 prevented amplification of randomly inserted template by annealing outside the repair template. Primers F1 and R1 were used to generate a 455 bp nested PCR amplicon for analyses by restriction enzyme digestion. **(C)** Restriction-digestion pattern of nested PCR amplicons from wild-type (WT) and edited lines after digestion with *Mme*I or *Bfa*I. + indicates positive control that includes the *Mme*I site and lacks the *Bfa*I site. **(D)** Vegetatively propagated edited line L1 with multi allele conversion of W574L and S653I was tolerant to application of the herbicide nicosulfuron (Accent® DuPont) at 95 g ha^−1^, in contrast to non-edited WT plants.

The initial screening of target mutations was completed *via* restriction enzyme (RE) digestion. Long PCR amplicons (1,913 bp) were generated with the primers DO1 and UP6 (Supplementary Table 3), the latter located outside (upstream) of the template sequence to prevent the amplification of randomly inserted templates. Primers F1 and R1 were used to generate shorter, 455 bp amplicons in a nested PCR for subsequent screening *via* RE digestion. The targeted mutation W574L is expected to introduce an *Mme*I RE recognition site. Mutation S653I is expected to eliminate the *Bfa*I RE recognition site (Figure 1). Enzyme digestion was performed following the manufacturer's instructions.

### TaqMan® Probe-Based Genotyping Assays

Intended mutations were further verified using fluorescent-labeled TaqMan® MGB probes, designed to detect wild-type and mutant alleles at mutation sites W574L and S653I in two HDR-genotyping assays (Supplementary Figure 3). Fluorophore VIC® was employed for wild-type alleles and fluorophore FAM was used for mutant alleles (Thermo Fisher Scientific, MA, USA). Thermal-cycling conditions constituted initial denaturation at 95°C for 10 min and 45 cycles of denaturation and extension at 95°C for 15 s and at 62°C for 1 min, respectively. The relative fluorescence units (RFUs) from probes were detected in a CFX Connect Real-Time PCR Detection System (Bio-Rad, CA, USA) and used to generate allelic discrimination plots. Sugarcane lines were assigned as events with intended mutations provided if RFU^mut^ > 200 and RFU^mut^/RFU^wt^ > 0.1 or if 200 > RFU^mut^ > 20 and RFU^mut^/RFU^wt^ > 0.4. RFU^mut^ and RFU^wt^ are mutant and wild-type allele RFU-values, respectively. Example discrimination plots in the HDR detection assay are displayed in Supplementary Figure 3.

### Amplicon Sequencing Using the Sanger Chain Termination Method

Cloned *ALS* alleles from transgenic plants with CRISPR/Cas9-mediated modifications were sequenced using the Sanger method. Briefly, long PCR amplicons (1,913 bp) generated with primers DO1 and UP6 (Supplementary Table 3) were cloned in pGEM®-T Easy Vector (Promega, WI, USA) and sequenced with M13 forward and reverse sequencing primers at Eurofins Genomics (Louisville, KY, USA). The sequences of 15–88 cloned PCR amplicons from *ALS* alleles of randomly selected events that were positive in TaqMan® assays for mutation sites W574L and/or S653I (Table 2) were also compared with the repair template to identify the presence of naturally occurring single nucleotide polymorphisms (SNPs; Table 3). This was done to evaluate the low probability of template switching between sugarcane *ALS* alleles and randomly inserted repair templates during PCR amplification and to distinguish between alleles.

**Table 2:** Edited sugarcane lines with intended mutations W574L and S653I.

	**TaqMan**^**®**^ **probe-based HDR-detection assay**				**Amplicon sequencing with Sanger chain termination method** ^‡^						
	Mutation frequency at W574 (%)^*^	**W574L^†^**	Mutation frequency at S653 (%)^*^	**S653I^†^**	**Total number of amplicons**	**Number of amplicons with intended mutations**				Mutation frequency at W574 (%)^**^	Mutation frequency at S653 (%)^**^
						**W574L**	**S653I**	**W574L and/or S653I**	**W574L and S653I**		
L1	21.6	+	46.9	+	88	27	47	69	5	30.7	53.4
L2	55.0	+	0	–	24	13	0	13	0	54.2	0
L3	1.7	–	25.7	+	23	0	6	6	0	0	26.1
L4	49.5	+	56.0	+	17	6	6	11	1	35.3	35.3
L5	41.8	+	47.2	+	17	5	7	13	0	29.4	41.2
L6	71.2	+	54.0	+	16	8	5	11	2	50.0	31.3
L7	0	–	67.3	+	15	0	5	5	0	0	33.3
L9	68.9	+	64.5	+	67	32	38	62	8	47.8	56.7
L10	45.5	+	45.1	+	24	9	14	21	2	37.5	58.3
L11	24.3	+	30.7	+	24	7	10	17	0	29.2	41.7
L13	68.9	+	0	–	22	15	1	16	0	68.2	4.5
L15	52.7	+	51.7	+	21	9	11	19	1	42.9	52.4
L16	48.5	+	38.0	+	22	8	8	16	0	36.4	36.4
L17	39.1	+	9.1	+	22	8	3	10	1	36.4	13.6
L18	22.0	+	30.8	+	23	6	8	14	0	26.1	34.8
L19	85.4	+	0	–	23	16	2	16	2	69.6	8.7
**Total**					**448**	**169**	**171**	**319**	**22**		
**Average**	**43.51**		**35.44**							**37.11**	**32.98**
WT	0	–	0	–	4	0	0	0	0	0	0

*Intended mutations were determined using a TaqMan® probe-based HDR-detection assay and amplicon sequencing, with the Sanger chain termination method*.

† *Presence (+) or absence (–) of intended mutations were determined according to allelic discrimination plots and relative fluorescent unit (RFU) values obtained using a TaqMan® probe-based HDR detection assay*.

‡ *PCR amplicons 1,913 bp in length were generated from genomic DNA from edited sugarcane lines and sequenced bi-directionally after cloning*.

* *Frequency was calculated as percentage of fluorescence signal from probe targeting the mutations W574L or S653I to total fluorescence signal*.

** *Frequency was calculated as percentage of amplicons with intended mutations W574L or S653I to total number of amplicons sequenced*.

**Table 3:** Naturally occurring SNPs distinguishing the different *ALS* alleles from the repair template in edited sugarcane *ALS* alleles.

**Nucleotide location**	**205**	**238**	**296**	**316**	**451**	**548**	**616**	**619**	**790**	**925**	**1,075**	**1,089**	**1,151**	**1,187**	**1,203**	**1,249 (PAM1)**	**1,317 (W574L)**	**1,483**	**1,513**	**1,554 (S653I)**	**1,628 (PAM2)**	**1,709**	**1,759**	**1,791**	**1,817**	**1,860**
Line and edited allele number																										
L1 allele 1	T	G	A	C		C			G									C		T	A				G	
L1 allele 2				C		C	T	C	G	A						T	T						T	A	G	C
L1 allele 3																T	T			T	A	T			G	
L4 allele 1				C		C	T	C	G			T				T	T			T	A	T			G	
L6 allele 1											C					T	T			T	A	T			G	
L6 allele 2				C		C	T	C					A			T	T						T	A	G	C
L10 allele 1																T	T			T	A	T			G	
L10 allele 2				C		C	T	C	G	A						T	T						T	A	G	C
L10 allele 3				C	T	C	T	C	G							T	T						T	A	G	C
L10 allele 4	T	G	A	C	T	C						T		C	G			C	T	T	A				G	
L15 allele 1																T	T			T	A	T			G	
L17 allele 1	T	G	A	C		C			G							T	T			T	A	T			G	
L19 allele 1				C		C	T	C	G			T				T	T			T	A	T			G	

*Intended edits introduced with HDR in ALS alleles are shown in red. Naturally occurring SNPs distinguishing the different ALS alleles from the repair template are shown in black. Analysis of edited ALS allele amplicons from seven selected lines (L1, L4, L6, L10, L15, L17, L19) and presence of naturally occurring SNPs in these amplicons suggests the absence of template switching by the DNA polymerase between sugarcane ALS alleles and the randomly inserted repair template during PCR amplification*.

## Results

### *In vitro* Cleavage Assay of Target DNA With Cas9 Nuclease Confirms sgRNA Activity

The amino acid substitutions in the *ALS* gene known to confer herbicide tolerance include W574L and S653I. The expression cassettes for two sgRNAs were designed and custom-synthesized to induce DSBs by Cas9 near these two codons. The two sgRNAs were tested *in vitro* with the commercially available Cas9 protein. The electrophoretic separation of *in vitro* cleavage products confirmed that both sgRNAs effectively targeted Cas9 to induce DSBs at the targeted sites in the *ALS* gene (Supplementary Figure 4).

### PCR Analyses of Sugarcane Events Following Co-transformation With Genome Editing Tools and Selectable Marker

The biolistic transfer of five combinations and ratios of unlinked selectable *npt*II marker and genome editing components was carried out as shown in Table 1 and in Supplementary Figures 1, 2. Following selection with Geneticin 467 independent callus lines were confirmed as PCR-positive for *npt*II (Table 1). Following selection with BS, 146 independent events (2–3 independent events per shot) were regenerated to plants (Table 1).

### Detection of Mutations by Altered Restriction Enzyme Digestion Patterns

The introduction of precision nucleotide substitutions at two codons associated with herbicide resistance and the corresponding PAM sites was targeted using a 1,833 bp double-stranded DNA repair template (Figure 1A, Supplementary Figure 1). This template differed from endogenous sugarcane *ALS* in four nucleotides, including two PAM sites and two targeted codons W574L and S653I. The PCR amplification of the target *ALS* amplicon from putatively edited sugarcane lines excludes the randomly inserted template using primer UP6, annealing to the *ALS* coding region upstream of the donor template (Figure 1A). Targeted mutation W574L introduces an *Mme*I RE recognition site, and mutation S653I eliminates a *Bfa*I site (Figure 1B). In contrast to the wild-type control, edited lines displayed both digested and undigested *ALS* amplicons (Figure 1C).

### Edited Lines Identified *via* TaqMan® Genotyping Assays

To allow for analyses of more than 100 edited events, a high-throughput TaqMan® genotyping assay was developed for the discrimination of DNA edits in the sugarcane ALS alleles as a result of HDR (Table 2, Supplementary Figure 3). For the HDR detection assays, the mutant probes were designed to recognize and bind sequences containing point mutations in the W574L or S653I codons and produce a signal. In the absence of DNA editing with HDR, the fluorescence signal was produced by the wild-type probe. The high level of polyploidy and variation in multi-allelic editing in sugarcane resulted in a range of fluorescent signals from different events. Representative allelic discrimination plots constructed with RFU from probes that detect wild-type or mutant alleles are presented in Supplementary Figure 3. These plots were used to identify edited plants with intended mutations. The number of sugarcane lines with intended mutations W574L and/or S653I are presented in Table 1. Frequencies of recovered lines with W574L and/or S653I mutations in treatments 1–5 ranged from 3.2 to 17.6% of the *npt*II positive callus lines prior selection with BS. The BS selection was not very stringent and more than 40% of the lines that regenerated on BS containing medium did not display edits. The frequency at which both mutations (W574L and S653I) were introduced into the same edited line ranged between 0 and 6.6% of the *npt*II positive callus lines prior selection with BS. The highest percentage of lines with both mutations was observed after treatment 2, where only sgRNA2 was delivered (Table 1). The lowest percentage of sugarcane lines with intended mutations was observed after treatment 5. No edited line carrying both mutations was recovered from this treatment. If a single sgRNA was used, editing was also observed at the distant target site 317 and 300 nt from the targeted DSB for W574L and S653I, respectively (Table 1).

### Sanger Sequencing of Cloned Amplicons Confirms Edited Lines Identified *via* the TaqMan® Genotyping Assay

**Figure 2:**
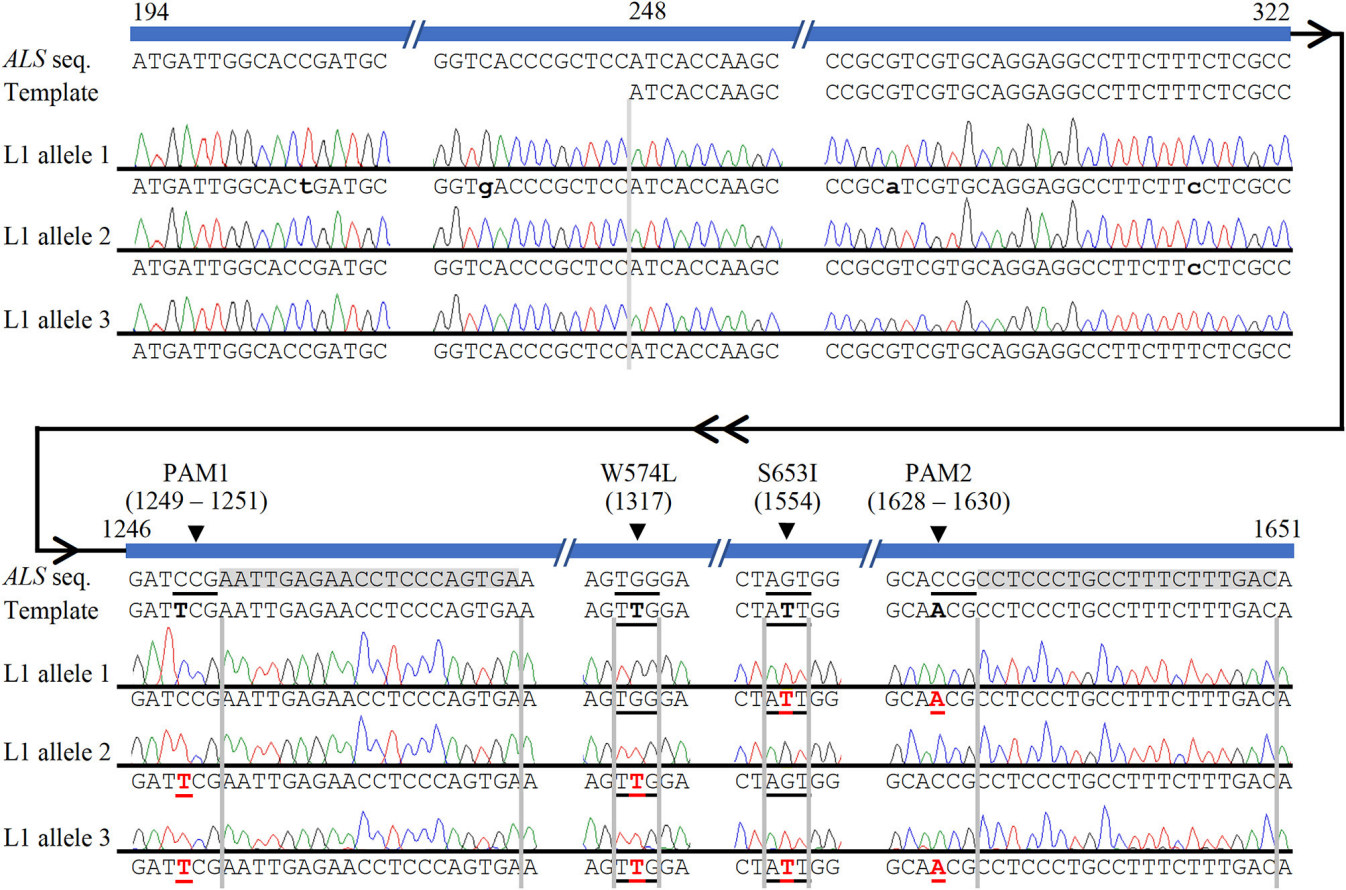
Confirmation of multi-allelic gene targeting in sugarcane by Sanger sequencing of cloned PCR amplicons from *ALS* alleles. The first 247 bp (194–247 bp shown here) of each cloned *ALS* amplicons were used to eliminate possible amplification and sequencing of donor template. Naturally occurring non-target single nucleotide polymorphisms, which were used to identify alleles, are indicated with lower case black letters. HDR-mediated CRISPR/Cas9 gene targeting edits are shown in red bold font and underlined. The four targeted nucleotide substitutions are highlighted in the template in black bold font for contrast with the wild-type *ALS* sequence. The two sgRNA sequences are highlighted in gray. HDR-mediated nucleotide substitutions of S653I and PAM2 (allele 1), W574L and PAM1 (allele 2), or both herbicide resistance sites and both PAM sites (allele 3) are shown in edited line L1. Numbering follows the 1,913 bp PCR amplicon of *ALS* alleles.

The high level of polyploidy in sugarcane (2*n* = 100 – 120) results in multiple copies/alleles of the *ALS* gene. Sanger sequencing and analysis of naturally occurring SNPs of cloned PCR amplicons of the *ALS* gene from non-modified sugarcane cultivar CP 88 – 1,762 allowed us to distinguish 23 sequence variants that represent different copies or alleles (Supplementary Table 4). Sanger sequencing of the cloned PCR amplicons was used for validation of the TaqMan® genotyping assay and to analyze *ALS* alleles with targeted precision nucleotide substitutions and naturally occurring SNPs. Sanger sequencing of 16 independent lines confirmed GT at the W574L and/or S653I codons in all 16 lines analyzed, which were selected according to positive TaqMan® probe-based HDR-detection assays (Table 2). For most lines, the frequencies of targeted nucleotide substitutions in amplicon sequencing corresponded to frequencies indicated by the TaqMan® HDR-detection assay. Co-editing with targeted nucleotide substitutions in three unique alleles was confirmed in one line analyzed by Sanger sequencing (Figure 2). Several lines including L5, L11, L16, and L18 displayed each of the targeted nucleotide substitutions in different alleles (W574L in one allele and S653I in another allele, Table 2). Sequence comparison of the PCR amplicons from edited *ALS* alleles with the repair template revealed the presence of naturally occurring SNPs in these amplicons (Table 3). When a single sgRNA was used (Treatment 1, Table 1), Sanger sequencing also confirmed editing at the distant S653I target and its corresponding PAM site, 300 or 374 nt from the targeted DSB in two independent lines (Supplementary Figure 5). In these two events, the PAM and W574L target site proximal to the DSB were not edited.

### Evaluation of Vegetative Progenies of Edited Sugarcane for Herbicide Resistance

Line L1, with multi-allelic precision nucleotide substitutions, was vegetatively propagated under greenhouse conditions to investigate herbicide resistance. Both wild-type plants and four edited sugarcane plants with multi-allele edits of the *ALS* gene were sprayed with nicosulfuron (Accent® DuPont) at the four-leaf stage with twice the labeled rate, 4 weeks after the stem cuttings were planted. One month after the application of herbicide, the wild-type sugarcane plants showed severe necrosis, in contrast to the edited plants, which had green leaves (Figure 1D). Then 2 months after herbicide application, all four edited plants were growing vigorously, whereas the wild-type sugarcane plants had died (data not shown).

## Discussion

Highly efficient and reproducible GT in sugarcane (*Saccharum* spp. hybrid) is reported here for the first time. Sugarcane has the highest genetic complexity of any crop, due to the interspecific origin of its highly polyploid (2*n* = 100–120) genome (D'Hont et al., 1996; Piperidis and D'Hont, 2020). Precision genome editing by HDR is an urgently needed tool for crop improvement (Huang and Puchta, 2019) and will support the targeted conversion of inferior to superior alleles. Template-mediated repair of DNA DSBs induced by the programmable nuclease CRISPR/Cas9 introduced multi-allelic precision nucleotide substitutions in the *ALS* gene of sugarcane, as confirmed by Sanger sequencing, conferring resistance to the herbicide nicosulfuron.

Precision genome editing of crops *via* HDR-mediated nucleotide substitutions has rarely been reported (Huang and Puchta, 2019). By contrast, loss-of-function mutations *via* the error-prone NHEJ repair pathway have been described in many crops (Yin et al., 2017; Huang and Puchta, 2019; Mao et al., 2019) including sugarcane (Jung and Altpeter, 2016; Kannan et al., 2018; Eid et al., 2021). Homology-directed repair frequency, as calculated as a percentage of GT events to all generated events, is typically in the range of 0.1 to a few percent (D'Halluin et al., 2013; Schiml et al., 2014; Svitashev et al., 2015; Endo et al., 2016; Hahn et al., 2018), which is more than an order of magnitude below the frequencies reported for NHEJ-mediated knock-outs of gene function (Yin et al., 2017; Hua et al., 2019; Huang and Puchta, 2019; Mao et al., 2019). This has been attributed to the superior efficiency of NHEJ for DNA repair in somatic cells (Puchta, 2005).

At least one targeted nucleotide substitution (W574L and/or S653I) in the *ALS* gene was detected in 54 of the 146 independent lines which regenerated from 467 transgenic callus lines in five different experiments. The HDR frequency for at least one targeted nucleotide substitution (W574L and/or S653I) in regenerated events ranged between 3.2 and 17.6% of the *npt*II positive lines prior BS selection. Plants with two resistance-conferring codons display a higher level of herbicide tolerance (Pozniak and Hucl, 2004; Shimizu et al., 2005). Two targeted nucleotide substitutions (W574L and S653I) were identified in 11 lines, accounting for 2.4% of the 467 lines prior BS selection.

Polymerase chain reaction artifacts from random head-to-tail inserts of the repair template and template switching of the DNA polymerase during PCR amplification have been described as potentially compromising the accurate evaluation of HDR frequency (Won and Dawid, 2017; Skryabin et al., 2020). The primers were designed to anneal to conserved regions for the amplification of all *ALS* alleles determined in this study. To prevent the PCR amplification of randomly integrated template DNA and selectively amplify the sugarcane *ALS* alleles, one of the chosen primers annealed to the 5′ *ALS* coding sequence, which was not part of the template. In addition, the sequence comparison of the PCR amplicons from the edited *ALS* allele with the repair template indicates the presence of naturally occurring SNPs in the PCR amplicons. This suggests the absence of template switching by the DNA polymerase between sugarcane *ALS* alleles and the randomly inserted repair template during PCR amplification. Evaluation of more than 400 bidirectional Sanger sequencing reads did not reveal any chimeric PCR amplicons representing random head-to-tail insertions of the repair template.

Double-strand breaks enhance recombination frequencies between homologous plant chromosomes or between sequence repeats (Puchta, 1998; Hayut et al., 2017; Taagen et al., 2020; Zhao et al., 2021). In diploid tomato, allele-dependent HDR reached 14% of all detectable DSB repair events (Hayut et al., 2017). The high polyploidy level in sugarcane may have contributed to the frequent occurrence of the HDR reported here. Twenty-three allelic variants of the ALS gene were identified by SNP analysis following Sanger sequencing of cloned PCR amplicons. The large number of target copies per cell may increase the chance that homologous recombination will occur. This provides a unique opportunity for crop improvement and genetic studies using GT.

Homology-directed repair efficiency in plants is highly correlated with the amount, size, and type of the repair template delivered to the target cells (Huang and Puchta, 2019). Biolistic gene transfer offers the ability to control the quantity of the delivered DNA during the particle coating reaction, influencing the frequency of GT and the number of transgene copies that are randomly inserted into the genome (Sandhu and Altpeter, 2008; Lowe et al., 2009; Sun et al., 2016). Elevating the molar ratio of DNA repair template four times over the other genome-editing components resulted in the highest HDR frequencies in this study. Recently, the use of geminiviral replicons as HDR vectors has increased the copy number of the template DNA delivered by *Agrobacterium tumefaciens* to target crops (Baltes et al., 2014; Butler et al., 2016; Wang et al., 2017). Elevating the template quantity with geminiviral replicons increased the HDR frequency to 34.5% in tetraploid potato (Butler et al., 2016) and to 25% in diploid tomato (Dahan-Meir et al., 2018). For HDR in sugarcane, the optimum template amount was 6 ng per kb of delivered repair template DNA. Doubling the delivered repair template quantity resulted in much lower editing efficiencies. Large numbers of randomly inserted repair template copies can lead to false-positive HDR results (Lawrenson et al., 2021). Our results from comparing the delivery of different repair template quantities suggest that excessive amounts of exogenously supplied DNA can have a negative impact on the repair process and that this did not inflate the HDR results in the developed assays.

The design of the repair template with long homology arms (Zhang et al., 2013) may also have contributed to the high HDR frequency (11.6% of the *npt*II positive callus lines and 36.9% of the lines that regenerated from BS medium on average of five experiments) in sugarcane reported here. Different repair outcomes with both W574L and S653I substitutions or one to four targeted nucleotide substitutions in one or multiple alleles were observed. Several lines displayed each of the targeted nucleotide substitutions in different alleles. The vast majority of targeted nucleotide substitutions at the W574L or S653I site occurred simultaneously with the corresponding proximal silent mutation of the specific PAM site. This supports the conclusion that the portion of the donor that is used in strand invasion, Holliday junction formation, and branch migration determines the outcome of HDR (Puchta and Fauser, 2015). Similarly, in rice, three haplotypes of HDR were observed at the S627 locus after a double-stranded DNA donor fragment was introduced (Sun et al., 2016). Sanger sequencing revealed multiplexed co-editing of up to three of the unique, cloned sugarcane *ALS* amplicons displaying the targeted nucleotide substitutions.

Editing was observed at both codon 574 and 653 at distances of 63 or 80 nt from the targeted DSBs. If a single sgRNA was used, edits were also observed 300 or 317 nt from the targeted DSB. Homology-directed repair repair frequencies are typically negatively correlated with distance to the DSB site (Baur et al., 1990; Paquet et al., 2016). The limiting step for HDR-mediated edits at more distant sites is likely 5′- to 3′-end resection. The factors involved in eukaryotic end resection have been thoroughly investigated in *Saccharomyces cerevisiae*, revealing that the number of DSBs per cell plays an important role in the activation of end resection. Four DSBs in yeast cells induced end resection of at least 300 nt in the G1 phase in 22% of the induced DSBs. In contrast, a single DSB resulted in only 8% of its induced DSBs, displaying an at least 300 nt end resection (Zierhut and Diffley, 2008). Single nucleotide polymorphisms analyses of *ALS* amplicons from non-edited sugarcane target cultivar CP 88–1762 suggest the presence of 23 *ALS* copies/alleles. The high number of *ALS* copies will result in an elevated frequency of DSBs per cell, potentially leading to more efficient end resection. This should enable both high HDR frequencies and the generation of HDR events distant from the cut site. However, co-delivering both sgRNAs instead of a single one did not elevate the frequency of the targeted nucleotide substitutions. Simultaneous cleavage at two sgRNA target sites can result in large deletions, conferring loss of function of the allele. This may have a deleterious effect on plant regeneration due to the essential role of *ALS* in the synthesis of branched chain amino acids in plants (Miflin and Cave, 1972). In addition, the use of two sgRNAs may elevate the frequency of NHEJ repair which may lead to a lower number of target sites that can be repaired using the donor template.

Surprisingly, the editing frequency was higher at the W574L codon than at the S653I codon, regardless of whether a sgRNA that cleaves 63 or 317 nt away from it was used. The 5′ homology arm of the repair template was more than twice as long as the 3′ homology arm and may have contributed to this outcome. In addition, herbicide selection in tissue culture and the major contribution of the W574L mutation to the level of herbicide resistance (Tan et al., 2006) may have also created a bias for the recovery of events with the W574L mutation.

Repair outcomes with the nucleotide edit only at a site that is further away from the DSB, leaving the closer one as WT, were also observed. This could be explained by the use of a fragmented template during the HDR-mediated repair process. DNA shearing is prominent with particle bombardment, generating a range of DNA fragments (Banakar et al., 2019). A fragmented repair template that is suitable for HDR-mediated repair would have a single homology arm. Single homology arms direct HDR in mammalian cells and are more prone to local repair with template switching (Basiri et al., 2017; Paix et al., 2017; Suzuki et al., 2019). This may favor the use of the NHEJ pathway to repair the gap on the side with no homology arm. The coupling of homologous and non-homologous repair mechanisms to preserve genomic integrity has been documented in mammalian cells (Richardson and Jasin, 2000).

Further improvements to multiplex precision nucleotide substitutions in sugarcane may be enabled by manipulating the competing DNA repair pathways (Even-Faitelson et al., 2011; Qi et al., 2013; Endo et al., 2016) or increasing HDR frequency by tethering the DNA repair template to the genome editing tool (Aird et al., 2018). Base editors and prime editors have emerged as alternative strategies to template-mediated HDR for precision nucleotide substitutions (Huang and Puchta, 2021). Relative to the template-mediated HDR employed in this study, base editors are limited by their narrow target range editing window of approximately 10 bp at the target site (Rees and Liu, 2018). Similarly, prime editors are limited by their template size (10 and 20 bp). In addition, the efficiency of prime editors varies greatly between target sites and the frequent generation of unintended indels is a concern (Huang and Puchta, 2021).

Multi-allelic precision nucleotide substitutions in sugarcane conferred herbicide tolerance. All progeny plants displayed herbicide resistance in their entire foliage suggesting the absence of chimerism. Introduction of herbicide resistance by GT will be a very effective tool for selecting events following the co-editing of multiple target genes. By comparison, conventional sugarcane breeding is complicated by poor male fertility, difficulty synchronizing the flowering time of the parental lines, poor seed germination, and the disruptive effects of meiotic recombination on the predictable performance of the progenies (Scortecci et al., 2012). This established protocol for targeted nucleotide substitutions allows fast and efficient introduction of the selected gene variants into elite sugarcane cultivars without crossing and associated linkage drag in support of crop improvement and genetic studies.

## Conclusions

We report efficient and reproducible GT in sugarcane, enabling precise co-editing of multiple alleles *via* template- and homology-mediated repair of DNA DSBs induced by the CRISPR/Cas9 programmable nuclease. This work will enable crop improvement by modifying inferior alleles through multiplexed GT.

## Data Availability Statement

The raw data supporting the conclusions of this article will be made available by the authors, without undue reservation. Sanger sequencing reads of cloned PCR amplicons of the ALS gene were submitted to NCBI with GenBank accession IDs MZ268741-MZ268818 (Supplementary Table 5).

## Author Contributions

FA conceived, designed, and managed the research project. MO carried out the construction of vectors carrying sgRNAs and confirmed their activity *via in vitro* cleavage assay, carried out sugarcane tissue culture and transformation, confirmed regenerated transgenic sugarcane lines by PCR, cloned PCR amplicons from edited sugarcane lines, and analyzed them using the restriction digestion assay, Taqman® genotyping, and Sanger sequencing. AA coordinated and carried out sugarcane tissue culture and transformation and contributed to the molecular characterization of genome-edited plants. RK contributed the template design and molecular characterization of genome-edited plants. AM developed the herbicide application protocol. MO and FA wrote the manuscript. All authors contributed to the article and approved the submitted version.

## Conflict of Interest

The authors declare that the research was conducted in the absence of any commercial or financial relationships that could be construed as a potential conflict of interest.

## Supplementary Material

Supplementary Figure 1 Plasmids carrying various genome-editing elements. Expression of codon-optimized *Cas9* and *npt*II were under transcriptional control of CaMV35S promoter. An intron from HSP70 was included between the promoter and coding sequence. Termination signals from AtHSP and CaMV35S were located downstream of co*Cas9* and *npt*II, respectively. sgRNA were placed under the control of U6 promoter from *Oryza sativa*. Donor template with nucleotide modifications to introduce W574L and S653I, and two modified PAM sites (PAM1 and PAM2) to prevent cleavage of the template by sgRNA1 or sgRNA2, were designed according to sugarcane *ALS* sequence. Homology arms are indicated with double-headed arrows. Vector components are not given to scale. *npt*II, neomycin phosphotransferase II; CRISPR, clustered regularly interspaced short palindromic repeats; *coCas9*, codon optimized CRISPR-associated gene 9; RNA; sgRNA, single guide RNA; *ALS*, acetolactate synthase; P_CaMV35S_, promoter of cauliflower mosaic virus 35S RNA; I_HSP70_, intron of 70 kDa heat-shock protein; T_CaMV35S_, terminator of cauliflower mosaic virus 35S RNA; T_AtHSP_, terminator of *Arabidopsis thaliana* heat-shock protein; NLS1, nuclear localization signal from SV40; NLS2, nuclear localization signal from nucleoplasmin; P_U6_, U6 promoter from *Oryza sativa*; PAM, protospacer adjacent motif; kb, kilobases.

Supplementary Figure 2 Sugarcane tissue culture, plant regeneration, and genetic transformation. **(A)** General outline of sugarcane tissue culture and genetic transformation. **(B)** Calli placed at the center of a Petri dish for bombardment. **(C)** Selection of transgenic calli expressing *npt*II on geneticin containing culture medium. **(D)** Regeneration of plantlets on culture medium supplemented with the herbicide byspyridbac sodium.

Supplementary Figure 3 High-throughput TaqMan® probe-based genotyping assay. **(A)** Schematic drawing of the TaqMan® probe-based genotyping strategy to detect HDR events. Large PCR amplicons (1,913 bp) were generated with primers DO1 and UP6, the latter located outside of the template sequence to prevent amplification of randomly inserted templates. Fluorescent labeled TaqMan® probes were designed to detect wild-type and mutant alleles (fluorophore VIC® for wild-type and fluorophore FAM for mutant allele) at both amino acid positions 574 and 653 in two HDR-detection assays. **(B)** Expected allelic discrimination plot in a probe-based HDR-detection assay. **(C,D)** Allelic discrimination plots constructed with relative fluorescent units from probes detecting wild-type or mutant alleles were used to identify edited plants with intended mutations. Sugarcane lines with targeted mutations W574L or S653I are indicated with green triangles whereas lines showing only wild-type allele signals are indicated with blue squares. Positive control plasmids (+) are shown with orange circles. No template control did not produce fluorescent signals.

Supplementary Figure 4 Electrophoretic separation of cleavage products after *in vitro* Cas9 nuclease assay. Cleavage with commercially available Cas9 protein confirmed that both sgRNAs (sgRNA_574_ and sgRNA_653_) effectively targeted Cas9 to induce double strand breaks at the target sites in the *ALS* gene. M, marker.

Supplementary Figure 5 Targeted nucleotide substitutions in the sugarcane *ALS* gene in lines L3 and L7, 300 and 374 nt from the DNA double strand break, respectively, targeted with a single gRNA as determined by Sanger sequencing of cloned PCR amplicons. Homology-directed repair-mediated CRISPR/Cas9 gene targeting edits are shown in red bold font and underlined. The four targeted nucleotide substitutions were highlighted in the template in black bold font compared to the wild-type ALS sequence. Naturally occurring non-target single nucleotide polymorphisms (SNPs), used to identify alleles, are indicated with lowercase black letters. The single sgRNA sequence used in this treatment (Treatment 1, Table 2) is highlighted in gray. Homology-directed repair-mediated nucleotide substitutions of S653I and PAM2 are shown in edited lines L3 and L7. Numbering follows the 1,913 bp PCR amplicon of *ALS* alleles.

Supplementary Table 1 Sequence of expression cassettes carrying gRNAs.

Supplementary Table 2 Sequence of sugarcane *ALS* gene and repair template.

Supplementary Table 3 Primer and probe sequences.

Supplementary Table 4 Sugarcane wild-type ALS alleles from CP 88–1762 determined with amplicon sequencing using the Sanger chain termination method.

Supplementary Table 5 GenBank accession IDs for sequence reads of cloned PCR amplicons of the ALS gene with intended mutations W574L and/or S653I from gene-edited lines.
