# Supplementary figures and images for: CRISPR/Cas9-Mediated Multi-Allelic Gene Targeting in Sugarcane Confers Herbicide Tolerance
Source: Front Genome Ed. 2021 Jul 8;3:673566. doi: 10.3389/fgeed.2021.673566 (PMC8525412; doi:10.3389/fgeed.2021.673566)

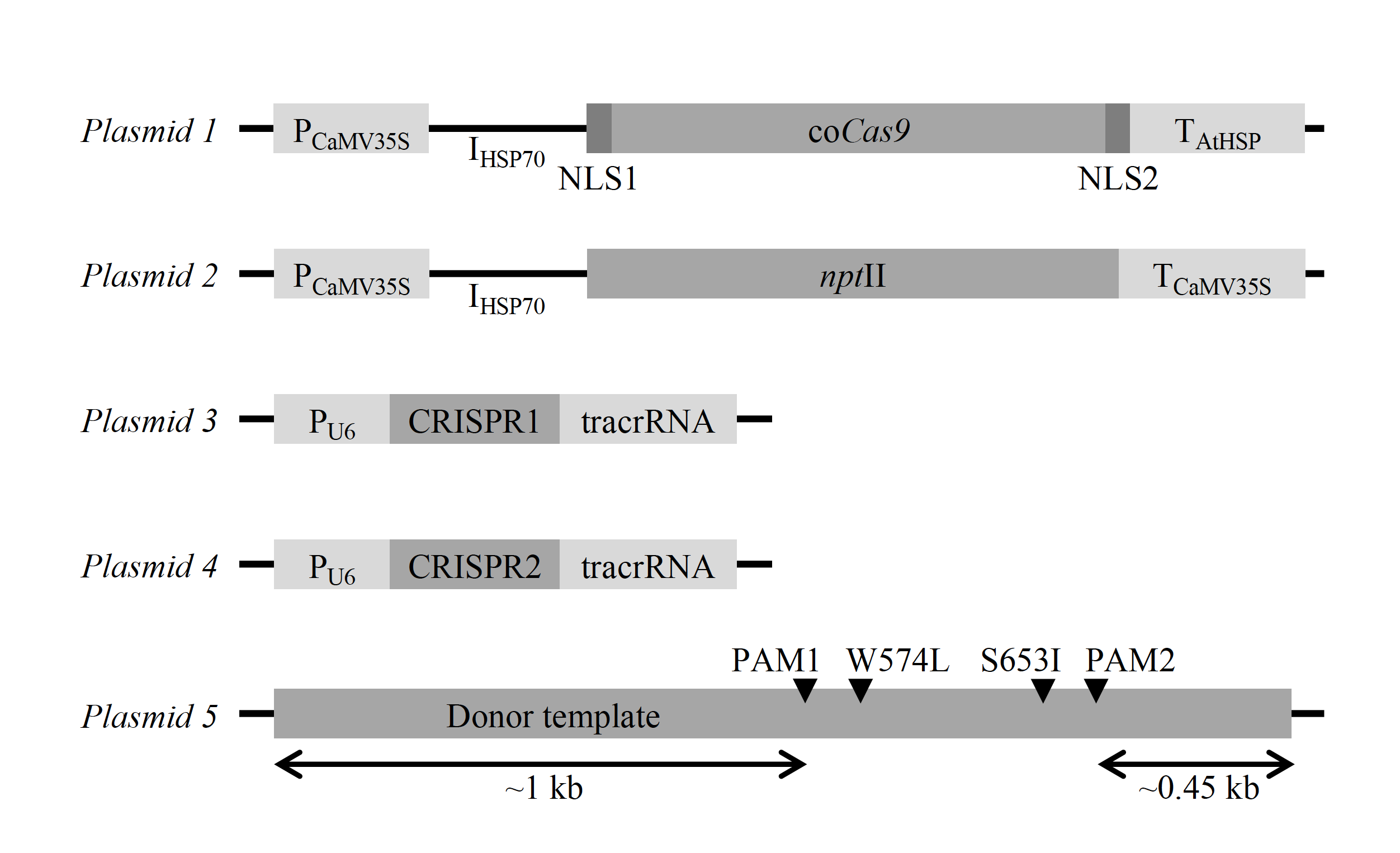

Supplement: Supplementary Figure 1 — Plasmids carrying various genome-editing elements. Expression of codon-optimized Cas9 and nptII were under transcriptional control of CaMV35S promoter. An intron from HSP70 was included between the promoter and coding sequence. Termination signals from AtHSP and CaMV35S were located downstream of coCas9 and nptII, respectively. sgRNA were placed under the control of U6 promoter from Oryza sativa. Donor template with nucleotide modifications to introduce W574L and S653I, and two modified PAM sites (PAM1 and PAM2) to prevent cleavage of the template by sgRNA1 or sgRNA2, were designed according to sugarcane ALS sequence. Homology arms are indicated with double-headed arrows. Vector components are not given to scale. nptII, neomycin phosphotransferase II; CRISPR, clustered regularly interspaced short palindromic repeats; coCas9, codon optimized CRISPR-associated gene 9; RNA; sgRNA, single guide RNA; ALS, acetolactate synthase; PCaMV35S, promoter of cauliflower mosaic virus 35S RNA; IHSP70, intron of 70 kDa heat-shock protein; TCaMV35S, terminator of cauliflower mosaic virus 35S RNA; TAtHSP, terminator of Arabidopsis thaliana heat-shock protein; NLS1, nuclear localization signal from SV40; NLS2, nuclear localization signal from nucleoplasmin; PU6, U6 promoter from Oryza sativa; PAM, protospacer adjacent motif; kb, kilobases. [file Image_1.TIFF]

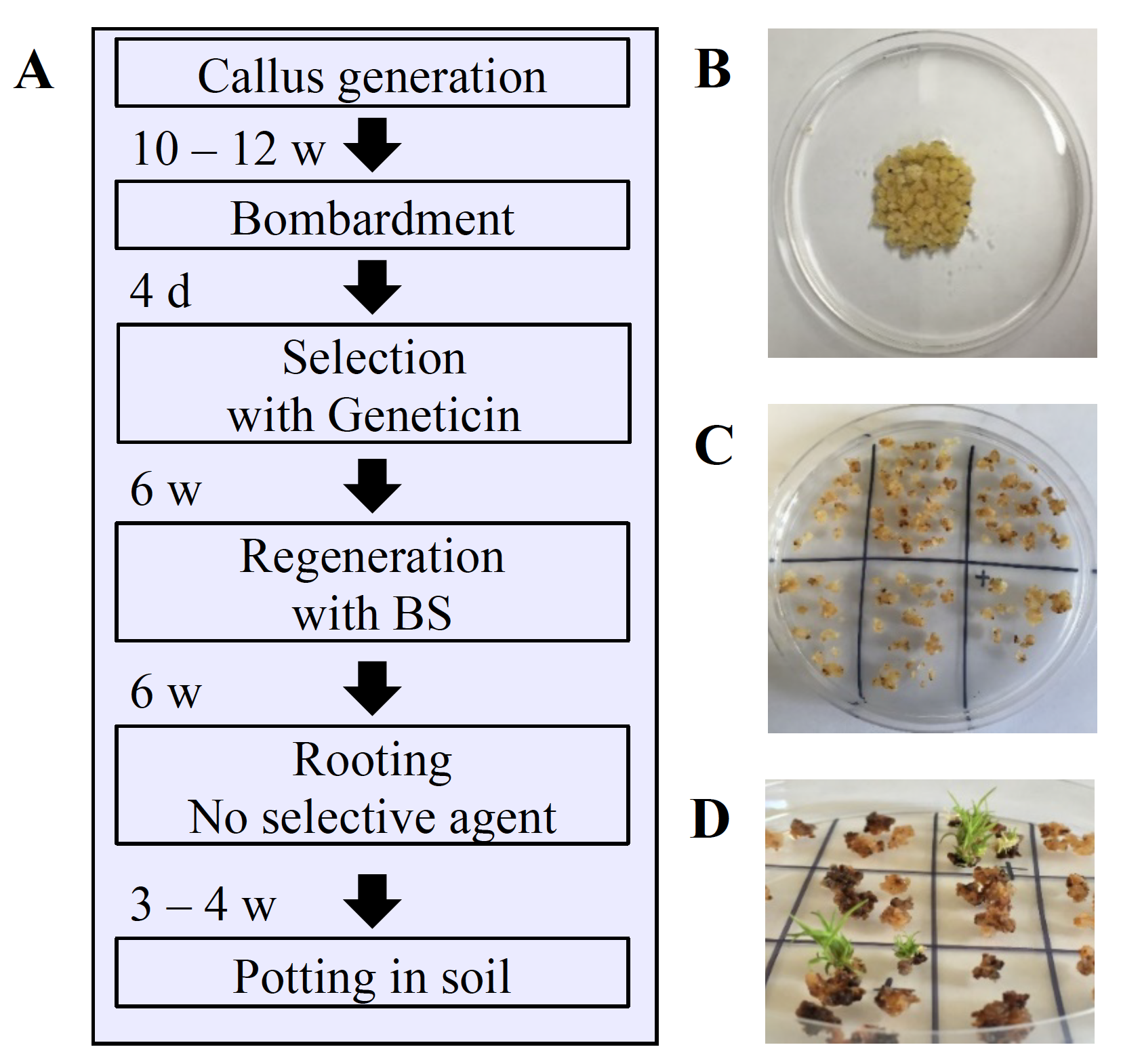

Supplement: Supplementary Figure 2 — Sugarcane tissue culture, plant regeneration, and genetic transformation. (A) General outline of sugarcane tissue culture and genetic transformation. (B) Calli placed at the center of a Petri dish for bombardment. (C) Selection of transgenic calli expressing nptII on geneticin containing culture medium. (D) Regeneration of plantlets on culture medium supplemented with the herbicide byspyridbac sodium. [file Image_2.TIFF]

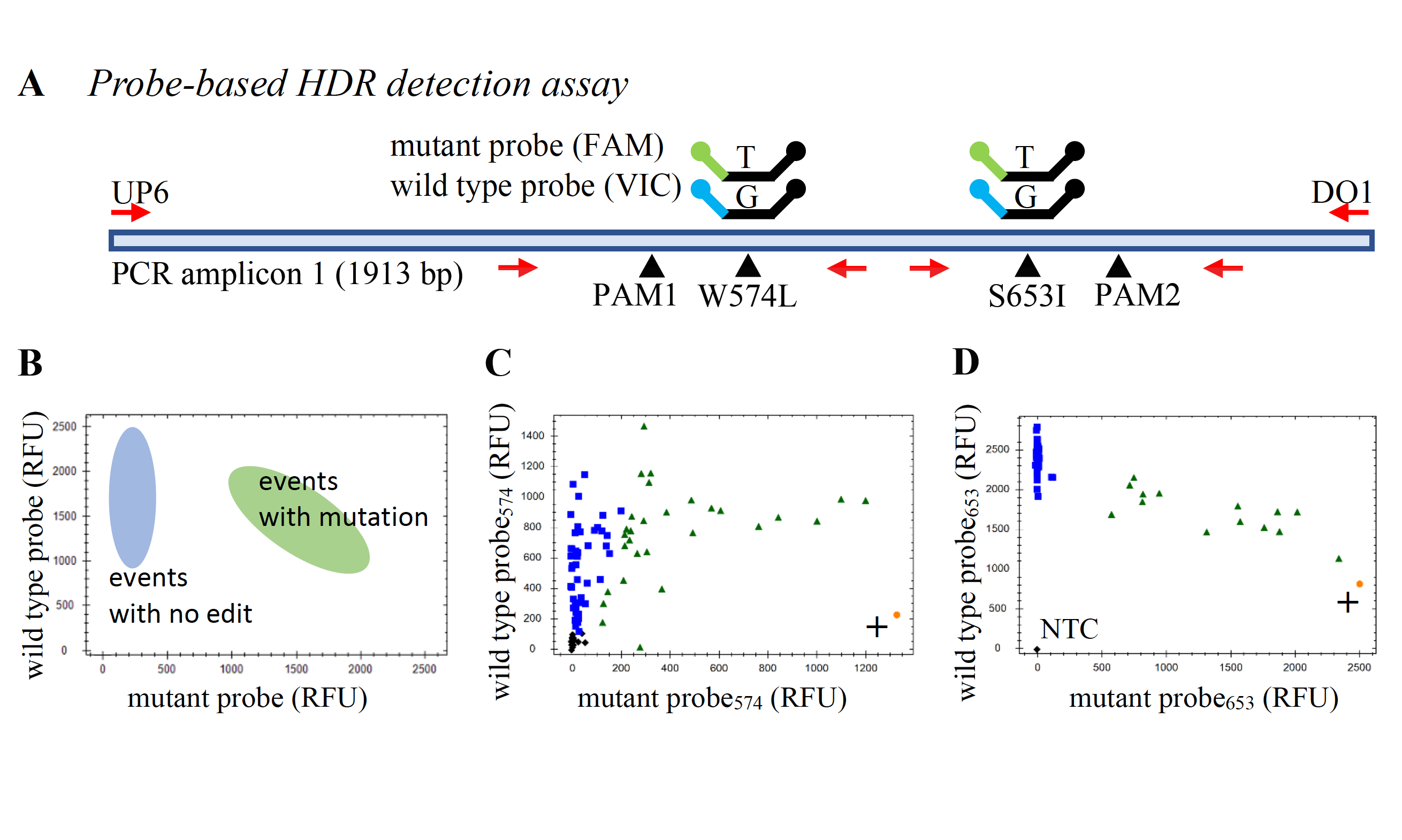

Supplement: Supplementary Figure 3 — High-throughput TaqMan® probe-based genotyping assay. (A) Schematic drawing of the TaqMan® probe-based genotyping strategy to detect HDR events. Large PCR amplicons (1,913 bp) were generated with primers DO1 and UP6, the latter located outside of the template sequence to prevent amplification of randomly inserted templates. Fluorescent labeled TaqMan® probes were designed to detect wild-type and mutant alleles (fluorophore VIC® for wild-type and fluorophore FAM for mutant allele) at both amino acid positions 574 and 653 in two HDR-detection assays. (B) Expected allelic discrimination plot in a probe-based HDR-detection assay. (C,D) Allelic discrimination plots constructed with relative fluorescent units from probes detecting wild-type or mutant alleles were used to identify edited plants with intended mutations. Sugarcane lines with targeted mutations W574L or S653I are indicated with green triangles whereas lines showing only wild-type allele signals are indicated with blue squares. Positive control plasmids (+) are shown with orange circles. No template control did not produce fluorescent signals. [file Image_3.TIFF]

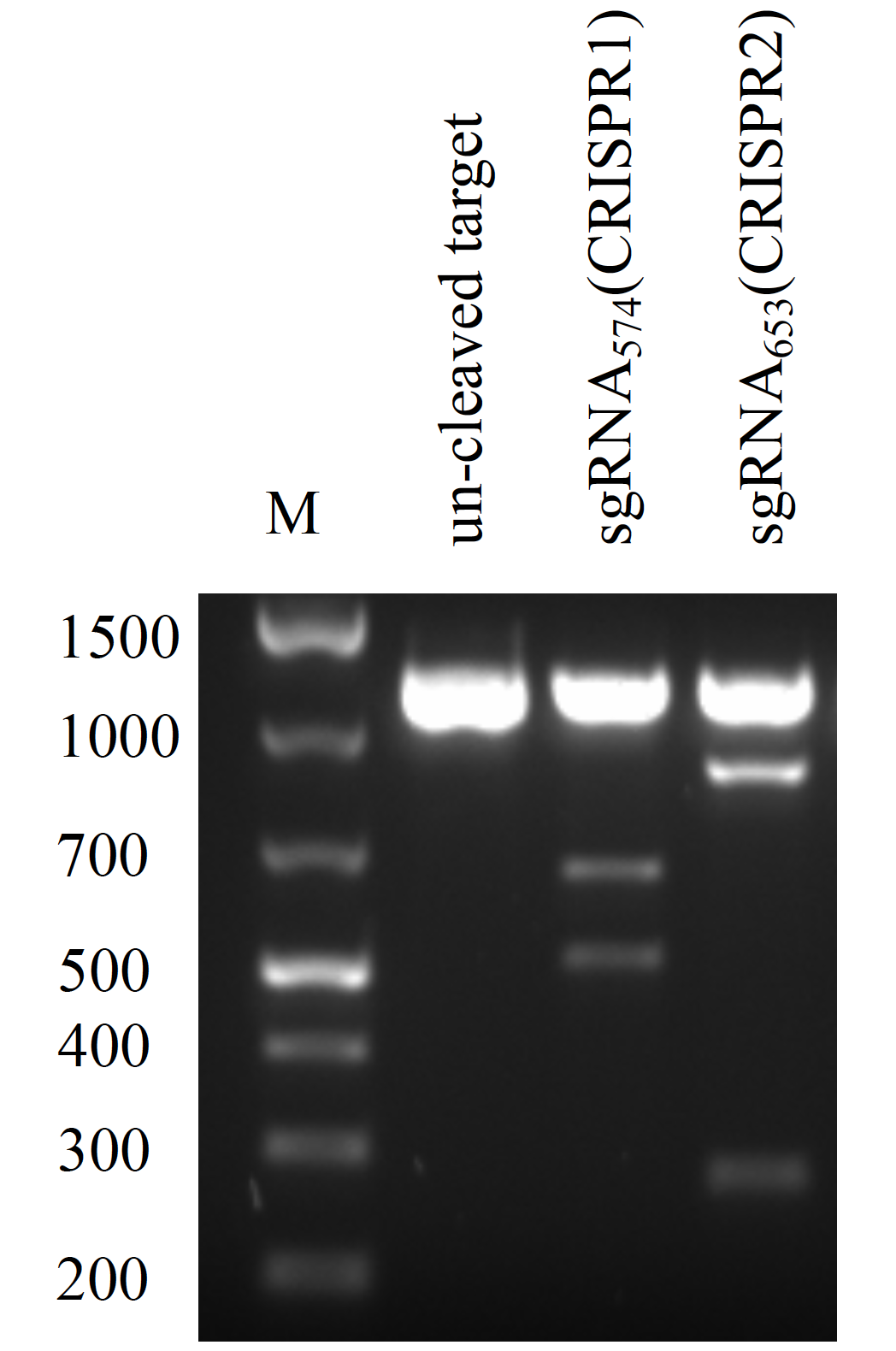

Supplement: Supplementary Figure 4 — Electrophoretic separation of cleavage products after in vitro Cas9 nuclease assay. Cleavage with commercially available Cas9 protein confirmed that both sgRNAs (sgRNA574 and sgRNA653) effectively targeted Cas9 to induce double strand breaks at the target sites in the ALS gene. M, marker. [file Image_4.TIFF]

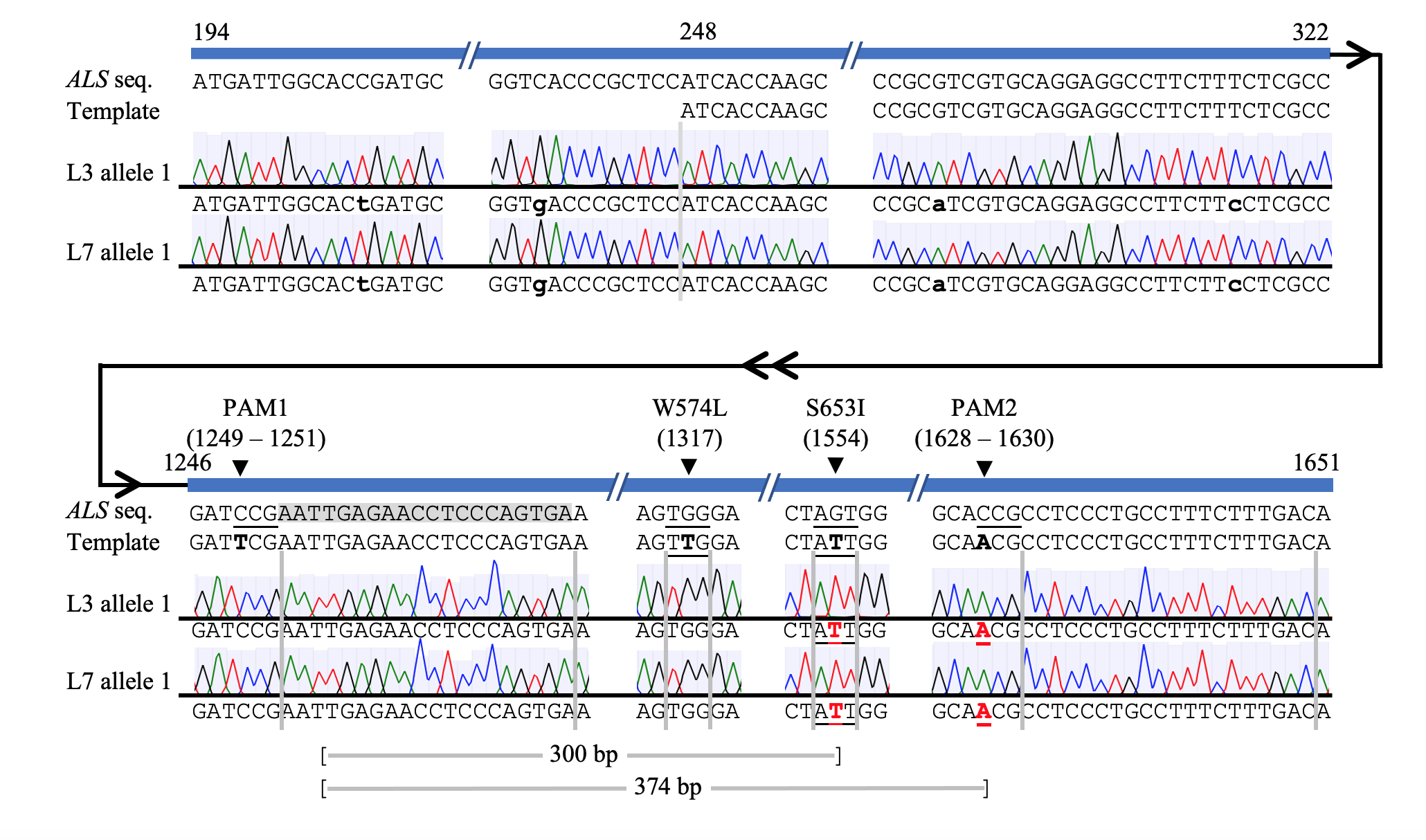

Supplement: Supplementary Figure 5 — Targeted nucleotide substitutions in the sugarcane ALS gene in lines L3 and L7, 300 and 374 nt from the DNA double strand break, respectively, targeted with a single gRNA as determined by Sanger sequencing of cloned PCR amplicons. Homology-directed repair-mediated CRISPR/Cas9 gene targeting edits are shown in red bold font and underlined. The four targeted nucleotide substitutions were highlighted in the template in black bold font compared to the wild-type ALS sequence. Naturally occurring non-target single nucleotide polymorphisms (SNPs), used to identify alleles, are indicated with lowercase black letters. The single sgRNA sequence used in this treatment (Treatment 1, Table 2) is highlighted in gray. Homology-directed repair-mediated nucleotide substitutions of S653I and PAM2 are shown in edited lines L3 and L7. Numbering follows the 1,913 bp PCR amplicon of ALS alleles. [file Image_5.TIFF]
