# Supplementary material for: CRISPR/Cas9-Mediated Multi-Allelic Gene Targeting in Sugarcane Confers Herbicide Tolerance
Source: Front Genome Ed. 2021 Jul 8;3:673566. doi: 10.3389/fgeed.2021.673566 (PMC8525412; doi:10.3389/fgeed.2021.673566)
Supplement: Supplementary Table 1 — Sequence of expression cassettes carrying gRNAs. [file Data_Sheet_1.pdf]

Supplementary Table 1. Sequence of gRNAs expression cassettes.

gRNA1 expression cassette. (Yellow highlight: U6 promoter; Green highlight: CRISPR1 targeting W574; Turquoise highlight: tracrRNA scaffold)	tttgtgaaagttgaattacggcatagccgaaggaataacagaatcgtttcacactttcgtaacaaaggtcttcttatcatgtttcagacgatggaggcaaggctgatcaaagtgatcaagcacataaacgcattttttttaccatgtttcactccataagcgtctgagattatcacaagtcacgtctagtagtttgatggtacactagtgacaatcagttcgtgcagacagagctcatacttgactacttgagcgattacaggcgaaagtgtgaaacgcatgtgatgtgggctgggaggaggagaatatatactaatgggccgtatcctgatttgggctgcgtcggaaggtgcagcccacgcgcgccgtaccgcgcgggtggcgctgctacccactttagtccgttggatggggatccgatggtttgcgcggtggcgttgcgggggatgtttagtaccacatcggaaaccgaaagacgatggaaccagcttataaacccgcgcgctgtagtcagcttgtcactgggaggttctcaattgttttagagctagaaatagcaagttaaaataaggctagtccgttatcaacttgaaaaagtggcaccgagtcggtgcttttttttttt
gRNA2 expression cassette. (Yellow highlight: U6 promoter; Green highlight: CRISPR2 targeting S653; Turquoise highlight: tracrRNA scaffold)	tttgtgaaagttgaattacggcatagccgaaggaataacagaatcgtttcacactttcgtaacaaaggtcttcttatcatgtttcagacgatggaggcaaggctgatcaaagtgatcaagcacataaacgcattttttttaccatgtttcactccataagcgtctgagattatcacaagtcacgtctagtagtttgatggtacactagtgacaatcagttcgtgcagacagagctcatacttgactacttgagcgattacaggcgaaagtgtgaaacgcatgtgatgtgggctgggaggaggagaatatatactaatgggccgtatcctgatttgggctgcgtcggaaggtgcagcccacgcgcgccgtaccgcgcgggtggcgctgctacccactttagtccgttggatggggatccgatggtttgcgcggtggcgttgcgggggatgtttagtaccacatcggaaaccgaaagacgatggaaccagcttataaacccgcgcgctgtagtcagcttgtcaaagaaaggcagggagggttttagagctagaaatagcaagttaaaataaggctagtccgttatcaacttgaaaaagtggcaccgagtcggtgcttttttttttt

Supplementary Table 2. Sequence of sugarcane *ALS* gene and repair template.

Coding sequence of *ALS*.<br>(Red font: Start codon and stop codon)	atggccaccaccgccgccgccgccgccgcgctcaccggcgccactaccgctgcgcccaaggcgaggcgccgggc<br>gcacctcctggccgcccggcgcgtcctcgccgcgcccatcaggtgctccgcggcgtcccccgccacgctgacggctccc<br>ccggccaccccgctccggccgtggggccccaacgagccccgcaagggcgccgacatcctcgtcgaggccctcgagcgc<br>tgcggcgttcgcgacgtcttcgcctaccccggcggcgcgtccatggagatccaccaggcactcacccgctcccccgtcatc<br>gccaaccacctcttccgccacgagcaaggggaggccttcgccgcctccggcttcgcgcgctcctcgggccgcgtcggcgt<br>ctgcgtcgccacctccggccccggcgccaccaacctagtctccgcgctcgccgacgcgctgctcgactccgtccccatggt<br>cgccatcacgggacaggtgccgcggcgcatgattggcaccgatgccttccaggagacgcccatcgtcgaggtcacccgct<br>ccatcaccaagcacaactacctggtcctcgacgtcgacgacatcccccgcgtcgtgcaggaggccttctttctcgcctcctct<br>ggtcgcccgggaccggtgcttgtcgacatccccaaggacatccagcagcagatggcggtgccggtctgggacacgcccat<br>gagtctgcctgggtacattgcgcgccttcccaagcctcctgcgactgaattgcttgagcaggtgctgcgtcttgttggtgaatc<br>gcggcgccctgttctttatgttggcggtggctgcgcagcatctggtgaggagttgtgccgctttgtggagatgactggaatccc<br>agtcacaactactcttatgggccttggcaacttccccggcgatgacccactgtctctgcgcatgcttggtatgcatggcacagt<br>gtatgcaaattatgcagtggataaggctgatctgttgcttgcatttggtgtgcggtttgatgatcgtgtgacagggaagattgag<br>gcttttgcaagcagggctaagattgtgcacattgatattgatccagctgagattggcaagaacaagcagccacatgtgtccatc<br>tgtgcagatgttaagcttgctttgcagggcatgaatgctcttctggaaggaagcacatcaaagaagagctttgactttggctcat<br>ggcacgatgagttggatcagcagaagagagaattccccttgggtataaacttttgatgaggagatccagccacagtatgct<br>atccaggttcttgatgagctgacaaaaggggaggccatcattgccacaggtgttgggcagcaccagatgtgggcggcacag<br>tactacacttacaagcggccaaggcagtggttgtcttcggctggtcttggggctatgggatttggtttgccggctgctgctggc<br>gctgctgtggccaacccaggtgtcactgttgttgacatcgacggagatggtagcttcctcatgaacattcaggagctagctatg<br>atccgaattgagaacctcccagtgaaggtctttgtgctaaacaaccagcacctggggatggtggtgcagtgggaggacagg<br>ttctataaggccaacagagcacacacatacttgggaaacccagagaatgaaagtgagatatatccagatttcgtgacaattgc<br>caaagggttcaacattccagcagtccgtgtgacaaagaagagcgaagtccatgcagcaatcaagaagatgcttgagactcc<br>agggccgtacctcttggatataatcgtcccgcaccaggagcatgtgttgcctatgatccctagtggtggtgctttcaaggatat<br>gatcctggatggtgatggcaggactgtgtattga
Repair template.<br>(**Red bold capital letters**: Introduced mutations; Underlined: First part of the template covering the CDS of *ALS* gene; *Italic*: Second part of the template covering the 3'UTR of *ALS* gene)	atcaccaagcacaactacctggtcctcgacgtcgacgacatcccccgcgtcgtgcaggaggccttctttctcgcctcctctgg<br>tcgcccgggaccggtgcttgtcgacatccccaaggacatccagcagcagatggcggtgccggtctgggacacgcccatga<br>gtctgcctgggtacattgcgcgccttcccaagcctcctgcgactgaattgcttgagcaggtgctgcgtcttgttggtgaatcgc<br>ggcgccctgttctttatgttggcggtggctgcgcagcatctggtgaggagttgtgccgctttgtggagatgactggaatcccag<br>tcacaactactcttatgggccttggcaacttccccggcgatgacccactgtctctgcgcatgcttggtatgcatggcacagtgta<br>tgcaaattatgcagtggataaggctgatctgttgcttgcatttggtgtgcggtttgatgatcgtgtgacagggaagattgaggctt<br>ttgcaagcagggctaagattgtgcacattgatattgatccagctgagattggcaagaacaagcagccacatgtgtccatctgtg<br>cagatgttaagcttgctttgcagggcatgaatgctcttctggaaggaagcacatcaaagaagagctttgactttggctcatggc<br>acgatgagttggatcagcagaagagagaattccccttgggtataaacttttgatgaggagatccagccacagtatgctatc<br>caggttcttgatgagctgacaaaaggggaggccatcattgccacaggtgttgggcagcaccagatgtgggcggcacagtac<br>tacacttacaagcggccaaggcagtggttgtcttcggctggtcttggggctatgggatttggtttgccggctgctgctggcgct<br>gctgtggccaacccaggtgtcactgttgttgacatcgacggagatggtagcttcctcatgaacattcaggagctagctatgat<br>**T**cgaattgagaacctcccagtgaaggtctttgtgctaaacaaccagcacctggggatggtggtgcagt**T**ggaggacaggtt<br>ctataaggccaacagagcacacacatacttgggaaacccagagaatgaaagtgagatatatccagatttcgtgacaattgcc<br>aaagggttcaacattccagcagtccgtgtgacaaagaagagcgaagtccatgcagcaatcaagaagatgcttgagactcca<br>gggccgtacctcttggatataatcgtcccgcaccaggagcatgtgttgcctatgatccta**T**tggtggtgctttcaaggatatg<br>atcctggatggtgatggcaggactgtgtattga*tctaaatttcagcaagca***A***cgcctccctgcctttctttgacatgcgcatg*<br>*agctagtacaagggtgataggtgttatctatgtgatggtctcctgtgttctatctttttgtaagccgtcagctatctatagtgtgc*<br>*ttgtctgatgtaccctgttatggtaatcttaagtagtctcctaccttgtagttgtgtagtctgttgttttcctgctggcatatctgtca*<br>*taagaggtcatgtaagtgccttttggtacacataaataaggtaataagcattgctatgcagtggttctgaattggcttctgttg*<br>*ccaaatttagttgtccaactggtccttgcttttgttttagctctttttccttgtttatttattattattgttaattccaactcaacatatg*<br>*atgtatggaaggatgcacatttgtgcatgaaagggctacttatatccaatttgttcttataagtatgttc*

Supplementary Table 3. Primer and probe sequences.

Identifier		Sequence (5’ to 3’)	Amplicon size	Purpose
OZ_NPTF	F	TACCTGCCCATTCGACCACC	345 bp	Verification of NPTII integration
OZ_NPTR	R	TAAAGCACGAGGAAGCGGTC		
UP6	F	CATCGCCAACCACCTCTTCC	1913 bp	Amplification of ALS gene fragment for sequencing
DO1	R	GAAGCCAATTCAGAACCACTGC		
F1	F	CTCATGAACATTCAGGAGCTAGC	455 bp	Amplification of ALS gene fragment for RE digestion
R1	R	ACCCTTGTACTAGCTCATGCGC		
UP5	F	ATTGAGGCTTTTGCAAGCAGGG	1174 bp	Amplification of ALS gene fragment for *in vitro* Cas9 cleavage assay
DO1	R	GAAGCCAATTCAGAACCACTGC		
C1F	F	GAATTTAATACGACTCACTATAGGG TCACTGGGAGGTTCTC	126 bp	Amplification of sgRNA1 (CRISPR1) with T7 promoter fused to 5’end
CR	R	AAAAAGCACCGACTCGG		
C2F	F	GAATTTAATACGACTCACTATAGGG TCAAAGAAAGGCAGGG	126 bp	Amplification of sgRNA2 (CRISPR2) with T7 promoter fused to 5’end
CR	R	AAAAAGCACCGACTCGG		
SoW574L_F	F	CGAATTGAGAACCTCCCAGTGAAG	137 bp	HDR detection assay for W574L
SoW574L_R	R	ATCTCACTTTCATTCTCTGGGTTTCC		
SoW574L_P1	P	TGCAGTGGGAGGACAG		TaqMan® probe for wild type allele (VIC® labeled)
SoW574L_P2	P	TGCAGTTGGAGGACAG		TaqMan® probe for mutant allele (FAM labeled)
SoW653L_F	F	GCACCAGGAGCATGTGTTG	82 bp	HDR detection assay for S653I
SoW653L_R	R	CACAGTCCTGCCATCACCAT		
SoW653L_P1	P	CCTATGATCCCTAGTGGTGG		TaqMan® probe for wild type allele (VIC® labeled)
SoW653L_P2	P	CCTATGATCCCTATTGGTGG		TaqMan® probe for mutant allele (FAM labeled)

F: Forward primer; R: Reverse primer; P: Probe; NPTII: Neomycin phosphotransferase II; CRISPR: Clustered regularly interspaced short palindromic repeats; coCas9: codon optimized CRISPR-associated protein 9; ALS: Acetolactate synthase; HDR: Homology directed repair; RE: Restriction enzyme; FAM and VIC® are fluorophores for probe labeling. VIC® is trademark of Life Technologies, Inc. TaqMan® is registered trademark of Roche Diagnostics GmbH.

Supplementary Table 4. Sugarcane wild-type ALS alleles from CP 88–1762 determined with amplicon sequencing using the Sanger chain termination method.

Nucleotide location	205	238	240	296	316	370	451	478	517	548	553	586	616	617	619	620	622	637	655	790	925	1241	1244	1276
Wild type allele no.																								
1	C	C	C	G	T	C	G	T	C	T	C	T	C	G	T	G	C	C	C	A	G	G	A	C
2	T	G		A	C		T			C					C	A	G			G				
3	T	G		A	C		T			C					C	A	G			G				
4	T	G		A	C		T			C					C	A	G			G				
5	T	G		A	C		T			C					C	A	G			G				
6	T	G		A	C																			
7			G	A	C		T			C					C	A	G			G				
8			G		C	T				C			T		C			G		G	A			
9			G		C	T				C			T		C			G		G	A			
10				A	C		T															A	T	A
11				A																				
12					C			A		C					C					G		A	T	A
13					C				T	C					C					G				
14					C					C			T		C					G				
15					C					C			T		C					G				
16					C					C			T		C					G				
17					C					C			T		C					G				
18					C							C		A	C				T	G				
19											A													
20																						A	T	
21																								
22																								
23																								

Nucleotide location	1348	1399	1453	1464	1483	1491	1574	1583	1629	1634	1635	1654	1656	1673	1751	1759	1791	1796	1797	1802	1816	1817	1860	1861
Wild type allele no.																								
1	A	C	C	C	T	C	A	G	C	C	C	C	C	G	T	C	T	G	T	T	C	C	G	T
2	G				C		T		T				T			T	A					G	C	
3	G				C					-	T		T	-		T		T	C			G		
4						T							T	-		T		T	C			G		
5									T				T	-		T		T	C			G		
6													T			T		T	C			G	C	C
7	G				C		T		T				T			T	A					G	C	
8		T							T				T									G	C	
9					C											T				C		G	C	
10			G		C								T		A	T						G	C	
11									T				T			T	A							
12			G		C								T		A	T						G	C	
13	G				C		T						A			T						G	C	
14	G				C		T		T				T			T	A					G	C	
15					C		T									T						G	C	
16					C			A								T				C		G	C	
17					C											T				C		G	C	
18												T	T			T						G	C	
19				A									T			T		T	C		A	G		
20													T	-		T		T	C			G		
21	G				C		T		T				T			T	A					G	C	
22	G				C		T						A			T	A					G	C	
23												T	T			T						G	C	

Allele 1 was used for template design. The template differs from allele 1 in its length and the 4 targeted nucleotide substitutions, including the W574L or S653I codons and silent mutations in the corresponding PAM sites. For consistency, nucleotide location numbering was done according to the 1913 bp long PCR amplicon used in analyses of *ALS* alleles in this study.

Supplementary Table 5. GenBank accession IDs for sequence reads of cloned PCR amplicons of the *ALS* gene with intended mutations W574L and/or S653I from gene-edited lines.

Line and edited allele no.	GenBank ID	Line and edited allele no.	GenBank ID
Saccharum hybrid spp. ALS	MZ268741	L10_allele03	MZ268780
L01_allele01	MZ268742	L10_allele04	MZ268781
L01_allele02	MZ268743	L10_allele05	MZ268782
L01_allele03	MZ268744	L10_allele06	MZ268783
L01_allele04	MZ268745	L11_allele01	MZ268784
L01_allele05	MZ268746	L11_allele02	MZ268785
L01_allele06	MZ268747	L11_allele04	MZ268786
L01_allele07	MZ268748	L11_allele06	MZ268787
L01_allele11	MZ268749	L12_allele01	MZ268788
L01_allele12	MZ268750	L12_allele02	MZ268789
L02_allele01	MZ268751	L13_allele01	MZ268790
L02_allele02	MZ268752	L13_allele02	MZ268791
L03_allele01	MZ268753	L14_allele01	MZ268792
L03_allele02	MZ268754	L14_allele02	MZ268793
L04_allele01	MZ268755	L14_allele03	MZ268794
L04_allele02	MZ268756	L14_allele05	MZ268795
L05_allele01	MZ268757	L14_allele06	MZ268796
L05_allele02	MZ268758	L15_allele01	MZ268797
L05_allele03	MZ268759	L15_allele02	MZ268798
L06_allele01	MZ268760	L15_allele03	MZ268799
L06_allele02	MZ268761	L15_allele04	MZ268800
L06_allele03	MZ268762	L15_allele05	MZ268801
L07_allele01	MZ268763	L16_allele01	MZ268802
L08_allele01	MZ268764	L16_allele02	MZ268803
L08_allele02	MZ268765	L16_allele03	MZ268804
L09_allele01	MZ268766	L16_allele05	MZ268805
L09_allele02	MZ268767	L16_allele06	MZ268806
L09_allele03	MZ268768	L17_allele01	MZ268807
L09_allele04	MZ268769	L17_allele02	MZ268808
L09_allele05	MZ268770	L17_allele03	MZ268809
L09_allele06	MZ268771	L17_allele05	MZ268810
L09_allele07	MZ268772	L17_allele08	MZ268811
L09_allele08	MZ268773	L18_allele01	MZ268812
L09_allele09	MZ268774	L18_allele02	MZ268813
L09_allele11	MZ268775	L18_allele04	MZ268814
L09_allele12	MZ268776	L18_allele05	MZ268815
L09_allele13	MZ268777	L19_allele01	MZ268816
L10_allele01	MZ268778	L19_allele02	MZ268817
L10_allele02	MZ268779	L19_allele03	MZ268818

ALS: Acetolactate synthase
